# Synthesis and Surface Engineering of Inorganic Nanomaterials Based on Microfluidic Technology

**DOI:** 10.3390/nano10061177

**Published:** 2020-06-17

**Authors:** Jie Shen, Muhammad Shafiq, Ming Ma, Hangrong Chen

**Affiliations:** 1 State Key Laboratory of High Performance Ceramics and Superfine Microstructures, Shanghai Institute of Ceramics, Chinese Academy of Sciences, Shanghai 200050, China; shenjie@student.sic.ac.cn (J.S.); hrchen@mail.sic.ac.cn (H.C.); 2 Center of Materials Science and Optoelectronics Engineering, University of Chinese Academy of Sciences, Beijing 100049, China; 3 Department of Chemistry, Pakistan Institute of Engineering & Applied Sciences (PIEAS), Nilore, Islamabad 45650, Pakistan; muhammad.shafiq917@yahoo.com

**Keywords:** microfluidics, inorganic nanomaterials, surface engineering

## Abstract

The controlled synthesis and surface engineering of inorganic nanomaterials hold great promise for the design of functional nanoparticles for a variety of applications, such as drug delivery, bioimaging, biosensing, and catalysis. However, owing to the inadequate and unstable mass/heat transfer, conventional bulk synthesis methods often result in the poor uniformity of nanoparticles, in terms of microstructure, morphology, and physicochemical properties. Microfluidic technologies with advantageous features, such as precise fluid control and rapid microscale mixing, have gathered the widespread attention of the research community for the fabrication and engineering of nanomaterials, which effectively overcome the aforementioned shortcomings of conventional bench methods. This review summarizes the latest research progress in the microfluidic fabrication of different types of inorganic nanomaterials, including silica, metal, metal oxides, metal organic frameworks, and quantum dots. In addition, the surface modification strategies of nonporous and porous inorganic nanoparticles based on microfluidic method are also introduced. We also provide the readers with an insight on the red blocks and prospects of microfluidic approaches, for designing the next generation of inorganic nanomaterials.

## 1. Introduction

Inorganic nanoparticles show prospect for an array of fields, such as imaging [1], optoelectronics [2], catalysis [3], sensing [4], and drug delivery [5], owing to their unique physicochemical properties at nanoscale. Despite significant progress in the synthesis and modification of inorganic nanomaterials, much effort needs to be made to establish different strategies, to accurately and precisely control the size of nanoparticles, as well as modulate their surface morphology and microstructure to meet specific goals and achieve the required functions.

By taking into consideration the physical state, the conventional bulk synthesis of inorganic nanomaterials can be classified into three methods: gas phase, liquid phase, and solid phase. Due to the lack of the accurate control of the mixing and separation process of reactants, the nucleation, growth, and agglomeration processes occur simultaneously in the conventional bulk synthesis method. Consequently, the structural, physicochemical, and functional properties of inorganic nanoparticles vary from batch-to-batch and nanostructures with the desired structure and features cannot be precisely achieved [6]. In addition, the insufficient mixing of reactants and unstable mass and heat transfer result into polydispersed and heterogeneous nanoparticles, with different physicochemical properties in the conventional bulk synthesis methods [7].

Microfluidics refers to the technology that integrates different fluids into a singular system in a specific way in micro-scale and precisely manipulates the behavior of microscale fluids (10^−9^ to 10^−8^ L) [8]. It provides new methods for many scientific and technological fields, including chemical synthesis, biochemical analysis, real-time monitoring of the chemical processes, disease diagnosis, drug development, and environmental monitoring. Microfluidic technology also plays a pertinent role in the synthesis and surface modification of inorganic nanomaterials. The electromechanical automation program of the microfluidic system can be used to accurately control the key factors affecting the structure and function of nanoparticles, such as total liquid flow, single-phase or multi-phase flow ratio, hydraulic pressure, and micro-reaction temperature [9]. The microfluidic reactor with high heat transfer efficiency can avoid the formation of large temperature gradients and can accurately control the reaction temperature. Moreover, since the flow time of the reactants in the microchannel is related to the channel length in the microfluidic synthesis, the reaction time or mixing time of different reactants can be varied by adjusting the channel length [10].

According to the reacting phases, the microfluidic methods can be divided into two categories: (1) single-phase continuous flow and (2) multiphase segmented flow [11]. The former one is performed with one or more miscible solvents, and the reagents are mixed by diffusion in laminar flow streams. Based on the structure of the channel, the single-phase continuous flow system includes T-type structure [12,13], Y-type structure [14], flow-focusing microchannel [15,16], and confocal microchannel [17]. On the other hand, the multiphase segmented flow is composed of at least two immiscible fluids: one a dispersed phase and the other a continuous phase [18]. By the addition of a new phase, the recirculation motion is triggered to cause the stretching and folding of the solution, thereby improving the fluid mixing efficiency. Meanwhile, it reduces the residence time of the reaction and overcomes the risk of channel blockage which may be caused by the direct contact between the liquid and the microfluidic channel. Since the additional reagents are usually difficult to be distributed uniformly into the already formed reaction droplets, it is difficult to realize the continuous reaction process of different reagents. It has been noticed that both single-phase continuous flow and multi-phase segmented flow microfluidic systems have the characteristics of the accurate manipulation of reaction parameters, efficient heat and mass transfer, large reaction interface, and good compatibility in real-time.

Due to the multiple characteristics and advantages of microfluidic technology as mentioned above, it has been widely used for the synthesis and surface modification of inorganic nanomaterials, such as silica nanoparticles [19], metal and metal/metal composite nanoparticles [20], quantum dots (QDs) [21], and metal-organic frameworks (MOFs) [22]. Additionally, the particle size, size distribution, and surface morphology/functionality of nanomaterials can be well controlled, and the batch-to-batch reproducibility can be further improved. More importantly, complex shapes and structures can also be realized by coupling multi-step synthesis and surface modification. In this review, the synthesis and surface modification of various types of inorganic nanoparticles using the unique capabilities of microfluidic devices have been systemically described. Meanwhile, their potential application in different sectors, including theranostics, therapeutics delivery, biosensing, and catalysis has been also briefly introduced.

## 2. Progress of Microfluidic Technology in the Synthesis of Inorganic Nanomaterials

Rapid nucleation followed by particle growth to the specific size without further nucleation can be realized by regulating a series of factors in the micron-scale confined space, resulting in the production of nanoparticles with much narrower particle size distribution than those obtained by bulk methods [23]. Different types of typical inorganic nanomaterials, such as silica-based nanomaterials, metal and metal composite nanomaterials, QDs, and MOFs have been successfully synthesized by employing microfluidic devices. The structural characteristics of microfluidic device, reaction mechanism and the application of each type of nanoparticle are presented in the following sections as well as summarized in Table 1.

### 2.1. Silica Nanomaterials

**Figure 1:**
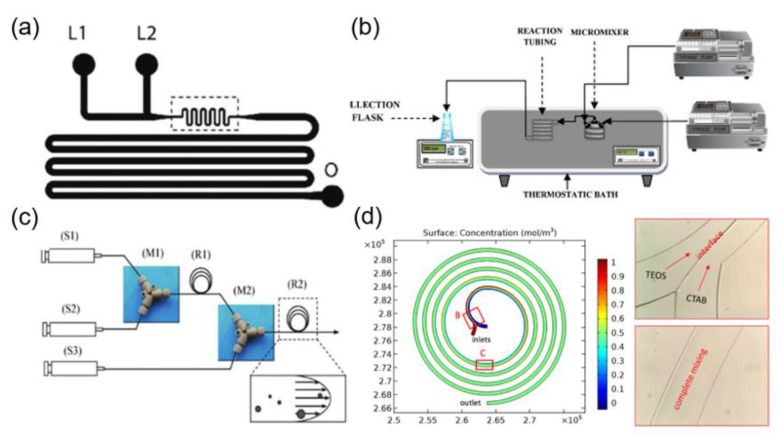
(**a**) Schematic illustration of microfluidic channels containing two liquid inlets (L1 and L2) and one outlet (O) for the preparation of silica nanoparticles (reproduced from [23], with permission from the American Chemical Society, 2004). (**b**) Experimental setup for the continuous synthesis of nanoparticle (reproduced from [24], with permission from the Elsevier, 2011). (**c**) Schematic diagram of microfluidic reactor systems with (S1) syringe for tetramethoxysilane (TMOS), (S2) syringe for 1 mM HCl, (S3) syringe for polyethylenimine (PEI) polymer in Tris–HCl buffer solution, (M1) and (M2) PEEK Y-shape mixers, (R1) reaction tube for hydrolysis, as well as (R2) reaction tube for silica precipitation (reproduced from [25], with permission from Elsevier, 2011). (**d**) Simulation and experimental results of reactants mixing in the spiral microchannel (reproduced from [26], with permission from Springer Nature, 2017).

The silica nanoparticles with the narrow size distribution and uniform structure can be obtained by microfluidic technology, due to the separation of nucleation and growth processes in the microfluidic system. For instance, Jensen and coworkers reported the synthesis of silica nanospheres by using a two-inlet type microfluidic chip (Figure 1a) [23]. In the synthetic process, the individual solutions containing silica precursors and alkaline catalysts were injected into separate inlets of the microfluidic chip. The results showed that the average particle size and particle size distribution could be controlled by adjusting the flow rate and residence time of the liquid phase. Yet, in another elegant study, it was demonstrated that the sequential injection of silica precursors into a micromixer and a capillary (1.3 mm internal diameter) Tygon^®^ pipe of variable length could result in the higher conversion rate of the reagent and narrow particle size distribution for the silica nanoparticles, in comparison with that of the bulk reactor system at a comparable reaction time (Figure 1b) [24]. However, it was found that silica nanoparticles were inevitably deposited within the microchannel, resulting in a certain degree of channel occlusion. It has been very recently demonstrated that the particle deposition within the microchannels can be significantly reduced by using commercial ethylene tetrafluoroethylene (ETFE) tubes (Figure 1c) [25], therefore enabling the continuous and scaled-up synthesis of silica nanoparticles.

Besides, mesoporous silica nanoparticles (MSNs) with tunable structures and morphologies have been synthesized by using microfluidic technology [26,27,28,29,30,31,71]. Zhang and coworkers synthesized MSNs with different morphologies by injecting liquid phases containing silica precursors and surfactants into two separate inlets in a spiral microfluidic chip (Figure 1d) [26]. The MSNs with different morphologies can be used for cell imaging, dye adsorption and drug delivery. Due to the effect of the transverse dean flow (resulted by the vortices occurring from fluid flow in curved channels), the two reactive liquid phases can be quickly and fully mixed. Consequently, the hollow silica nanospheres were obtained in a short time by optimizing the flow rates and mixing time of the reactants.

In addition to the spherical nanoparticles, non-spherical silica nanomaterials, including hollow ellipsoids, fibers, flowers, tablets, and triangles have been synthesized and proven to exhibit good biological behavior, which included high cell binding efficiency, transmembrane penetration, and long-term in vivo circulation [72,73,74,75,76,77]. However, there are still many outstanding challenges that need to be addressed to achieve the stable and controllable synthesis of non-spherical silica nanomaterials by using the conventional bulk synthesis approach. In contrast, in the microfluidic droplet-assisted synthesis of silica nanoparticles, the deformation or disassembly of each individual droplet can be well controlled through the design of complex microscale shape of the chip, resulting in the synthesis of non-spherical silica nanoparticles with different morphologies. For example, the aspect ratio of silica nanorods can be easily controlled by varying the ratio of the droplet volume to the cylindrical microchannel diameter [78]. Meanwhile, a series of the other non-spherical shaped silica materials, such as doughnut [79], raspberry [80], filbert [81], and disk [82] have also been obtained by microfluidic droplet-assisted synthesis.

In addition, the microfluidic template method has been exploited to synthesize non-spherical mesoporous silica nanomaterials. Mesoporous silica fibers were prepared by injecting cetyltrimethylammonium bromide (CTAB)/diluted ammonia solution and diluted tetraethyl orthosilicate (TEOS) into the two inlets of the microfluidic chip, which could be used for DOX drug loading and 4-nitrophenol reduction [30]. The aspect ratio and diameter of silica fiber could be adjusted by changing the flow rate and the concentration of reactants. Besides, in the same microfluidic device, two-dimensional mesoporous silica nanowires with a hollow sandwich-like bilayer structure and a water-ripple-like wrinkled surface were prepared by using CTAB/tetrabutylammonium iodide (TBAI) mixed surfactant as a template [47]. Yet, in another study, the anisotropic hollow ellipsoid-like mesoporous silica nanomaterials were produced within a few seconds by the rapid mixing of the pre-synthesized ellipsoidal MSNs and phosphate buffered saline (PBS) as an etching agent in microfluidic devices [31]. In contrast, the conventional bulk synthesis approach requires approximately a full day for chemical etching to obtain the similar type of hollow structures. The chemical etching strategies based on microfluidic devices can also be feasible for the synthesis of the other type of anisotropic silica nanomaterials, with hollow structures such as cubes, rods, and bands [32].

**Figure 2:**
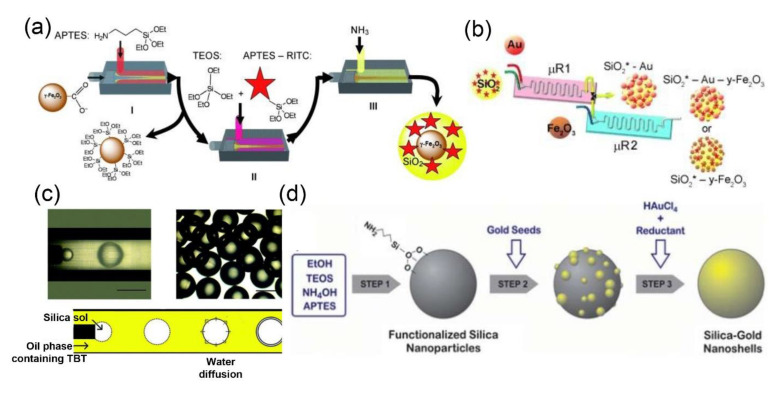
(**a**) Scheme for the continuous synthesis of core-shell structured Fe_2_O_3_-SiO_2_ nanoparticles (reproduced from [86], with permission from the Royal Society of Chemistry, 2009). (**b**) A two-step microfluidics-based synthesis procedure for the assembly of multifunctional SiO_2_-Au-Fe_2_O_3_ nanoparticles (reproduced from [87], with permission from the American Chemical Society, 2013). (**c**) Micrographs of the droplets and the mechanism of the synthesis of core–shell structured particles (reproduced from [85], with permission from the Royal Society of Chemistry, 2011). (**d**) Experimental process and set-up for the continuous synthesis of SiO_2_-Au nanoshells (reproduced from [88], with permission from the Royal Society of Chemistry, 2012).

Microfluidic methods can also be used for the preparation of silica-based core-shell nanocomposites [33,34,37,38,39,40,83,84,85]. Cabuil et al. carried out three-step reactions, including grafting, mixing, and coating, in a series of microfluidic devices to obtain multistage core-shell Fe_2_O_3_-SiO_2_ composites with fluorescence and magnetic properties, for in vivo fluorescence imaging and magnetic resonance imaging (MRI) (Figure 2a) [86]. Similarly, SiO_2_-Au-Fe_2_O_3_ nanostructures with fluorescence, plasma, and superparamagnetic functions were synthesized by two series glass microreators, containing two inlets and one outlet that can operate independently or in synergy (Figure 2b) [87].

Microfluidic technology can also be used to prepare silica-based hybrids with specific structure and desired properties, by accurately controlling the emulsification process to produce monodispersed compound droplets within the microchannel [40,85]. For instance, Luo and coworkers synthesized core-shell structured SiO_2_@TiO_2_ hybrids, by using a one-step emulsification process in a coaxial microfluidic system (Figure 2c) [85]. Meanwhile, core-shell structured SiO_2_-Au nanoparticles were synthesized by using a simple and scalable microstructure mixer (Figure 2d) [88]. Similarly, silica nanocomposites modified by gold nanoparticles have been synthesized by using a central collision microreactor [89]. Moreover, TiO_2_-SiO_2_, Fe_3_O_4_-SiO_2_-Pt, and Co_3_O_4_-SiO_2_ nanoparticles have also been fabricated by microfluidic technology [31,90,91,92].

**Figure 3:**
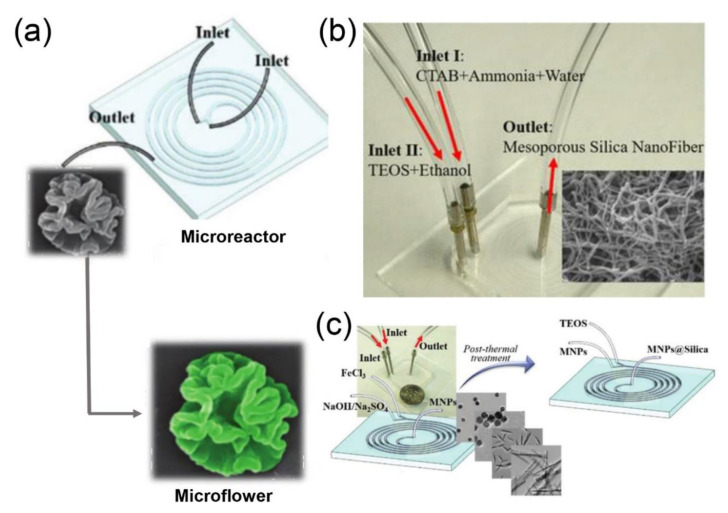
(**a**) Schematic illustration for the microfluidic synthesis of silica nanoflowers (reproduced from [93], with permission from the Royal Society of Chemistry, 2018). (**b**) Experimental setup for the microfluidic synthesis of silica fibers (reproduced from [30], with permission from the American Chemical Society, 2018). (**c**) Microfluidic synthesis of Fe_2_O_3_-SiO_2_ nanomaterials for circulating tumor cell screening (reproduced from [32], with permission from the Royal Society of Chemistry, 2018).

Recently, Hao and coworkers demonstrated the design and microfluidic fabrication of the silica nanoparticles with different morphologies (e.g., hollow spheres [26], nanoflowers (Figure 3a) [93], and fibers (Figure 3b) [30] containing different functional agents (e.g., fluorescent dyes, proteins, QDs, magnetic nanoparticles, and silver nanoparticles)). More recently, this group has also successfully coated the surface of magnetic nanoparticles with the silica shell layer through a spiral microreactor, to obtain core-shell structured Fe_2_O_3_-SiO_2_ nanocomposites with different shapes (Figure 3c) [32]. They indicated that the thickness of the outer shell can be well adjusted by changing the flow rate of the TEOS solution.

### 2.2. Metal and Metal Composite Nanomaterials

Metal or metal composite nanomaterials are widely used in catalysis [55], sensing [94], tumor therapy [95], energy technology [96], and several other fields, owing to their excellent physical and chemical properties. The heat and mass transfer processes play a decisive role in the nucleation and growth of metal nanoparticles. With an increase in the size of the reactor or the concentration of reagents, the heat transfer and mixing efficiency of the solution system becomes complicated and difficult to be accurately controlled. Precipitation is one of the most commonly used conventional bulk synthesis methods to obtain metal or metal composite nanomaterials [97]. In order to obtain particles with the small size and narrow size distribution, the reaction system needs to have high supersaturation; however, due to the limitation of heat and mass transfer, the high supersaturation of the whole system cannot be maintained during spontaneous nucleation. Therefore, it is cumbersome to obtain large quantities of metal or metal composite nanomaterials with high quality, narrow size distribution, and monodispersity. Compared with the conventional bulk synthesis approaches, the high specific surface area of the microfluidic channel renders microfluidic systems amenable for optimal heat and mass transfer [98]. The high heat transfer ability can reduce the temperature fluctuations, which may be caused by the endothermic or exothermic reactions. Concomitantly, the high mass transfer efficiency can shorten the reaction time and improve the yield, thus leading to a higher batch-to-batch reproducibility and high production efficiency.

#### 2.2.1. Microfluidic Synthesis of Metal Nanoparticles

The physical and chemical properties of metal nanomaterials are closely related to their microscopic particle size and morphology. Boutonner and coworkers pioneered the microfluidic synthesis of metal nanoclusters, including platinum, palladium, rhenium, and iridium in 1982 [99]. Subsequently, many researchers have synthesized various types of metal nanomaterials by using microfluidic technology, including gold, nickel, silver, and other metal nanoparticles.

Gold nanoparticles are widely used in various fields, such as disease diagnostics [100,101,102], photothermal therapy [103,104,105], drug delivery [106,107], and optical applications [108]. Recently, different types of synthetic strategies based on microfluidic technology have been reported for the structural manipulation and functionalization of gold nanoparticles. Gold nano-leaflets with ultra-thin folded structures were synthesized at room temperature by using a three-channel microfluidic chip [43]. In this microfluidic synthesis, cetyltrimethylammonium chloride (CTAC) and sodium borohydride (NaBH_4_) were used as protective and reducing agents, respectively. The gold nanosheets with thickness ranging from less than 1 to several nanometers could be obtained by adjusting the flow rate.

Multi-functional gold nanoparticles can also be fabricated in a microfluidic device. Functionalized gold nanoparticles have been synthesized by Santamaria and coworkers [44]. Different stages of the reaction process, including the activation of precursors, formation and growth of seeds, and the coating of polyethylene glycol (PEG) on gold nanoparticles, were integrated in a continuous single-phase microfluidic system, fully realizing the automated synthesis and functionalization of gold nanoparticles. Huang and coworkers synthesized shape-controllable tetrathiafulvalene (TTF)-Au composite nanoparticles, by employing a three-dimensional microfluidic chip [109]. As the flow rate of acetonitrile buffer was increased, the TTF-Au hybrids exhibiting different morphologies were achieved, which included branching aggregates consisting of irregularly-aligned polyhedral crystals, multilayered structures consisting of thin and flat layers, two-dimensional dendritic nanostructures, and coral-like aggregates consisting of fibers.

**Figure 4:**
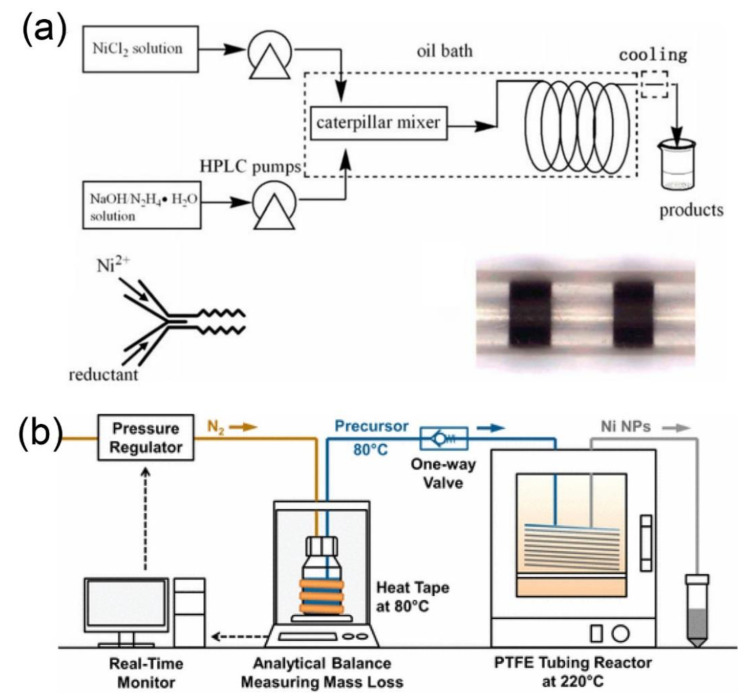
(**a**) Schematic illustration of the micro-reaction process for synthesis of crystalline nickel nanoparticles using an oil bath-heated capillary-type mixer (reproduced from [45], with permission from the Elsevier, 2012). (**b**) Reactor system for the continuous flow production of nickel nanoparticles (reproduced from [46], with permission from the American Chemical Society, 2017).

High-quality and scale-up production of nickel nanoparticles is critical to the industrial development, because these nanoparticles have important applications in catalysis and magnetic materials [110,111]. Zhang and coworkers employed a high performance liquid chromatography (HPLC) pump to inject the mixture of nickel chloride (NiCl_2_), hydrazine hydrate, and sodium hydroxide in an oil bath-heated capillary-type mixer, to obtain crystalline nickel nanoparticles with an average particle size of 80 nm and particles size distribution in the range of 60–114 nm (Figure 4a) [45]. The obtained nickel nanoparticles possess great application prospects for the catalytic hydrogenation of p-nitrophenol to p-aminophenol. The yield of this reaction is about 11.5 g h^−1^, which is much higher than that of conventional bulk methods. Yet, in another elegant study, a single-phase flow type microfluidic reactor was used to synthesize nanocrystalline nickel particles, and the flow rate of the precursor solution was measured by using an analytical balance in real time (Figure 4b) [46]. The computer control system was used to adjust the driving pressure of the precursor solution through the feedback loop, to ensure a constant flow rate during the operation of the microfluidic equipment. The mixture of nickel acetate (Ni(acac)_2_), oleylamine, trioctylphosphine, and 1-octadecene precursors could nucleate rapidly after entering into the microfluidic chamber at 220 °C. The average diameter of nickel nanoparticles and the yield were found to be 11.1 ± 3.1 nm and 62%, respectively. Moreover, it was estimated that more than 27 g of nickel nanoparticles could be produced in one day.

**Figure 5:**
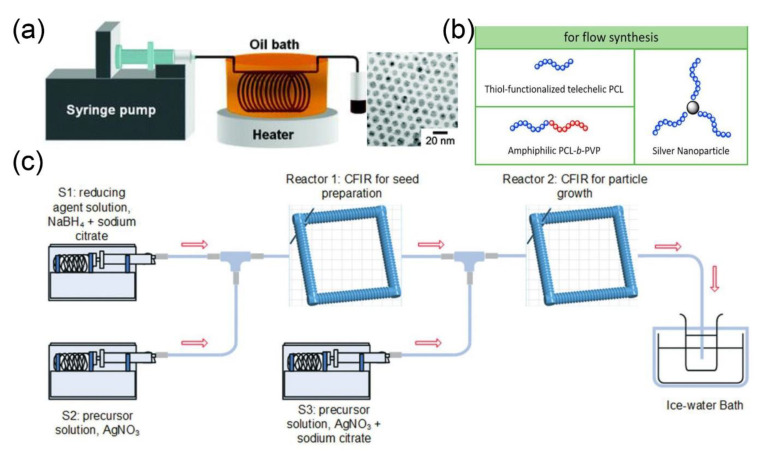
(**a**) Experimental setup for the synthesis of silver nanoparticles in a tubular-shaped microreactor and TEM images of silver nanoparticles (reproduced from [47], with permission from the American Chemical Society, 2004). (**b**) Chemoselective polymerization platform for the synthesis of polyester-modified silver nanoparticles (reproduced from [116], with permission from the Elsevier, 2018). (**c**) Experimental setup for fabrication of silver nanoparticles by using microfluidic step-by-step reaction strategy in a microfluidic chamber (reproduced from [49], with permission from the Royal Society of Chemistry, 2018).

Silver nanomaterials are well-known for their interesting properties and wide applicability in catalysis [112], optics [113], and biomedicine [114,115]. Due to a strong correlation between the structure of silver nanoparticles and their properties, the controlled fabrication of such nanoparticles has recently attracted the intensive attention of researchers worldwide. Silver nanoparticles were synthesized through a single-flow tubular type microreactor by using silver pentafluoropropionate as a precursor (Figure 5a) [47]. Similarly, anisotropic silver nanoparticles were prepared by the reduction of silver nitrate with NaBH_4_ in a microfluidic reactor, consisting of three different types of micromixers [48]. Moreover, Guo and coworkers constructed a microfluidic platform for the continuous-flow chemoselective polymerization, to synthesize polyester-modified silver nanoparticles (Figure 5b) [116].

Similarly, silver nanoparticles of different sizes (≤5 ± 0.8, 6.5 ± 1.2, 6.8 ± 1.2, 7.7 ± 1.4, 8.0 ± 1.5, and 9.3 ± 1.8 nm) were prepared by using a step-by-step reaction strategy in microfluidic technology (Figure 5c) [49]. The Dean number of the reactor determined the size and polydispersity of the nanoparticles. The higher Dean number (>6) produces smaller-sized silver nanoparticles (<6 nm), while the lower Dean number produces larger silver nanoparticles (>8 nm) with higher dispersity.

**Figure 6:**
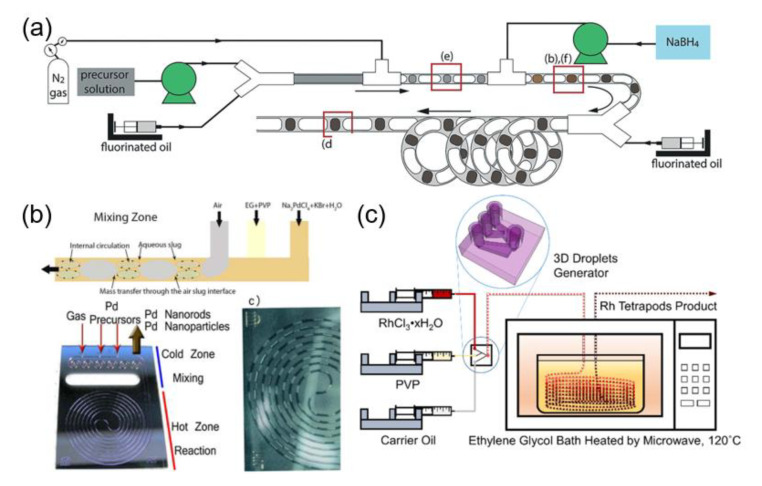
(**a**) Schematic illustration of the triphasic segmented flow reactor for the synthesis of ultra-small palladium nanoparticle (reproduced from [50], with permission from the Royal Society of Chemistry, 2017). (**b**) Schematic illustration of the segmented flow generated in the mixing zone of a hydrophilic microfluidic reactor (reproduced from [51], with permission from the Wiley-VCH Verlag GmbH & Co, 2016). (**c**) Schematic illustration of the two-phase flow microfluidic system for fabrication of rhodium tetrapod product (reproduced from [52], with permission from the American Chemical Society, 2017).

Precious metal nanoparticles, such as palladium and rhodium, can also be synthesized by microfluidic technology. Corbos et al. synthesized palladium nanoparticles in a single-channel flow reactor with a high production rate of about 10 L of nanoparticles suspension per day [50]. The designed reactor can separate nucleation and growth processes through an ingeniously designed microfluidic chip. The first reactor section provides the requisite intensified mixing for rapid nanoparticle nucleation, while the second section of the reactor allows the nucleated nanoparticles to age and grow to the particle of the desired diameter. Meanwhile, it avoids the reflux risk of hydrogen produced by the decomposition of NaBH_4_ (Figure 6a). The blockage of the channel has been reduced, as the fluorinated oil and nitrogen completely isolate the water phase from the channel wall. Moreover, the diameter of the microfluidic channel in the second stage of reaction is larger than that of the first stage, which helps to reduce the pumping pressure and achieve a higher production of nanoparticles.

A multiphase segmented flow microfluidic system has also been used to fabricate palladium nanoparticles (Figure 6b) [51]. It was established that the reaction temperature, dissolved oxygen, and potassium bromide (KBr) played important roles in controlling the morphology of palladium nanoparticles. Interestingly, isotropic nanospheres or anisotropic nanorods could be obtained by judiciously adjusting the residence time and flow rate. A two-phase flow microfluidic system was also employed to prepare rhodium nanoparticles. Different morphologies of nanoparticles were realized by varying the temperature and residence time (Figure 6c) [52]. At low temperature (120 °C) and short residence time (4.6 min), most of the nanoparticles exhibited multipod-like morphology. In contrast, most of nanoparticles were of cuboctahedral-shape at higher temperature (150 °C) and prolonged residence time (35 min). The reactor was made of (polytetrafluoroethylene) PTFE tubing, which could withstand a high temperature (260 °C).

#### 2.2.2. Microfluidic Synthesis of Metal Composite Nanoparticles

**Figure 7:**
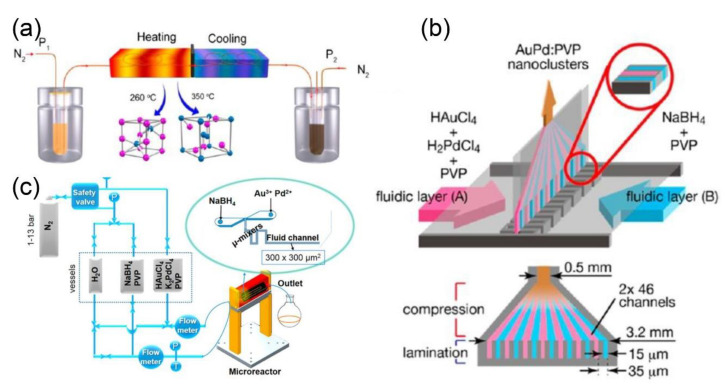
(**a**) Schematic representation of the microfluidic reactor system for the fabrication of Pt-Bi nanoparticles (reproduced from [53], with permission from the American Chemical Society, 2015). (**b**) Schematic illustration of the microfluidic mixing device for the synthesis of pure and bimetallic nanoparticles (reproduced from [54], with permission from the American Chemical Society, 2014). (**c**) Schematic illustration of the microfluidic setup for synthesis of polyvinyl pyrrolidone (PVP)-stabilized monometallic and bimetallic nanoparticles (reproduced from [55], with permission from the American Chemical Society, 2018).

The preparation of intermetallic compounds with uniform size and high purity usually requires high temperature and pressure in the conventional bulk approach, which is relatively more challenging as compared to single metal nanomaterials [117,118,119]. Guo and coworkers pioneered a new type of single-phase microfluidic reaction system to afford the synthesis of intermetallic compounds (Figure 7a) [53]. The microfluidic reaction device employed nitrogen to drive the precursor solution containing chloroplatinic acid (H_2_PtCl_6_), bismuth nitrate (Bi(NO_3_)_3_), and ethylene glycol into the channels. It was demonstrated that the phase and morphology of nanoparticles can be tuned by increasing the heating. Typically, V-shaped Pt_1_Bi_1_ nanostructures with an arm diameter of approximately 17 nm were obtained at 260 °C. On the other hand, spherical-shaped Pt_1_Bi_2_ nanostructures with an average diameter of 33.5 nm were synthesized at 360 °C. Meanwhile, bimetallic Au-Pd nanoclusters were prepared by using a single-phase multilayer flow mixing device for the catalytic aerobic oxidation of benzyl alcohol (Figure 7b) [54]. The rapid diffusion mixing between laminar flows was found to be beneficial for the synthesis of bimetallic nanostructures with smaller average particle size (1–3 nm). Likewise, a series of gold- and palladium-based nanoparticles, including Au, Au_7_Pd_3_, Au_5_Pd_5_, Au_3_Pd_7_, and Pd, were synthesized by varying the composition of metal precursors (Figure 7c) [55]. The obtained nanoparticles showed smaller size and narrow particle size distribution. The size of Au_7_Pd_3_ and Au_3_Pd_7_ nanoparticles were found to be 1.4 ± 0.5 nm and 1.6 ± 0.8 nm, respectively.

In addition to the single-phase microfluidic reaction system as mentioned above, the multiphase microfluidic reaction system has also been widely used for the synthesis of metal composites. In a recent study, octahedral-shaped Pt-Ni nanoparticles were fabricated by using a two-phase microfluidic reaction system [56]. Precursor solutions, including platinum(II) acetylacetonate (Pt(acac)_2_), Ni(acac)_2_ and tungsten hexacarbonyl (W(CO)_6_) were afforded in a mixture of oleic acid, oleylamine and benzyl ether and preheated to 70 °C before introduction into the microfluidic reaction system heated at 230 °C. In this process, W(CO)_6_ decomposes to immediately release carbon monoxide (CO) gas, which may (1) separate the liquid phase into uniformly distributed discrete droplets and (2) act as a reducing agent for the synthesis of Pt-Ni nanoparticles. In addition, by using air as a continuous phase and sodium tetrachloropalladate (II) (Na_2_PdCl_4_) and PVP dispersed diethylene glycol solution as a dispersed phase, the palladium icosahedra nanoparticles coated with platinum monolayer were obtained [57]. These core-shell type Pt-Pd nanomaterials can significantly improve the catalytic activity of platinum while being used as an oxygen reduction reaction catalyst.

#### 2.2.3. Microfluidic Synthesis of QDs

QDs are a class of semiconductor nanocrystals with photocatalytic [120,121], photoelectric conversion [122], and electrical properties [123], which possess wide-spread applications. Currently, there are two main synthetic methods for the synthesis of QDs: (1) vapor-phase epitaxial growth method and (2) liquid-phase method [124]. The vapor-phase epitaxial growth requires complex synthesis and it is also difficult to isolate QDs from the substrate [125]. It has also been elucidated that the function of QDs can be improved by ligand exchange in the liquid phase method; however, the slow mixing rate of precursor solution, low productivity, and poor reproducibility limit the application of this method for the scale-up production of QDs [126]. In contrast to the conventional bulk method, microfluidic technology has advantages of high mass and heat transfer rate, accurate and controllable reaction conditions, and low consumption of raw materials, which make it an ideal method for the scale-up synthesis of QDs [127]. Consequently, various types of QDs have been explored based on microfluidic technology in recent years.

**Figure 8:**
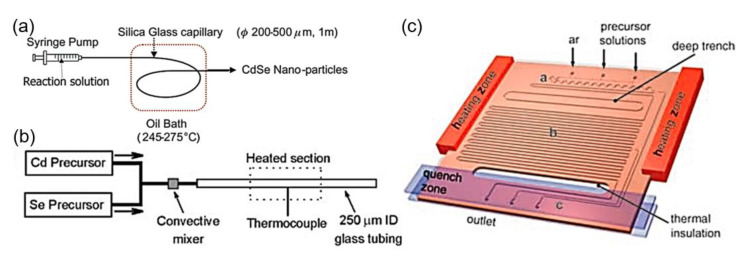
(**a**) Diagram of the flow reactor for the synthesis of CdSe nanoparticles (reproduced from [58], with permission from the Royal Society of Chemistry, 2002). (**b**) Schematic illustration of the capillary reactor for synthesis of CdSe nanocrystals (reproduced from [59], with permission from the Wiley-VCH Verlag GmbH & Co, 2003). (**c**) Schematic illustration of the reactor with heating zone and injected with gas for the synthesis of CdSe QDs (reproduced from [60], with permission from the Wiley-VCH Verlag GmbH & Co, 2005).

Cadmium-based QDs are promising candidates for tunable photoluminescence and high quantum yield [128]. Nakamura and group reported the preparation of cadmium nanocrystals by infusing cadmium acetate precursor solution into an oil bath-heated quartz glass capillary (Figure 8a) [58]. The particle size of the synthesized nanoparticles was found to be 2–4.5 nm and the absorption peak was observed in the range of 450–600 nm. In addition, cadmium selenide (CdSe) nanocrystals were synthesized by using a continuous phase microfluidic device (Figure 8b) [59]. A series of CdSe nanocrystals with different luminescence colors was synthesized by controlling the ratio of the precursors and reaction temperature. In another study, the gas was used as a buffer layer to separate precursor droplets, to shorten the reaction time and precisely control the particle size of CdSe QDs (Figure 8c) [60].

In addition, core-shell structures composed of two or more semiconductor materials, such as CdSe/ZnS QDs, can also be fabricated by microfluidic technology. A two-step microfluidic method has been adopted to afford core-shell CdSe@ZnS nanoparticles. The precursor solution was pumped into the first reactor to obtain the CdSe core, which was then followed by the mixing of CdSe core and ZnS precursor solution in the second reactor (Y-type microfluidic chip) to coat the ZnS layer on CdSe core [129]. To endow the CdSe@ZnS nanocrystals with the luminescence properties (fluorescence quantum yield of about 50% and full width at half-maximum of about 32 nm), the microfluidic device was upgraded [61]. Consequently, QDs with complex structures, such as core-shell multi-layer CdSe@CdS@ZnS nanocrystals were prepared by employing a multi-step sequential reaction, which showed a higher quantum yield (60%) as compared to CdSe@ZnS [62].

**Figure 9:**
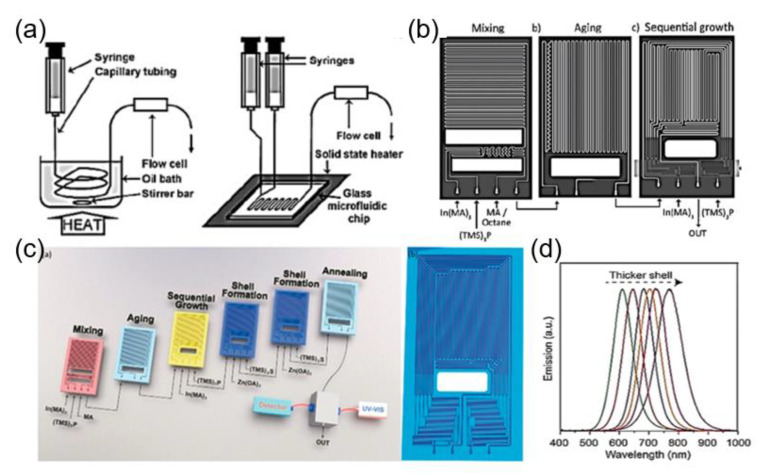
(**a**) Schematic illustration of single-capillary and Y-shaped microfluidic devices for the synthesis of InP QDs (reproduced from [63], with permission from the Wiley-VCH Verlag GmbH & Co, 2009). (**b**) Schematic illustration of the microfluidic reactor having three functions, including mixing, aging, and sequential injection (reproduced from [64], with permission from the Wiley-VCH Verlag GmbH & Co, 2011). (**c**) Illustration of a multistage microfluidic platform for the synthesis of core-shell InP/ZnS QDs. (**d**) Emission spectra of the InP/CdS QDs (reproduced from [67], with permission from the Wiley-VCH Verlag GmbH & Co, 2018).

Indium phosphate (InP) QDs with low cytotoxicity are considered as potential substitutes for Cd-based QDs, which show a great potential for versatile biomedical applications [130]. Nightingale and coworkers reported the synthesis of InP QDs based on a two-in/one-out y-shaped reactor, which provided precise control over the reaction temperature, the reaction time, and the flow rates (Figure 9a) [63]. Jensen and coworkers developed a microfluidic reactor, in which three stages of reaction, such as, mixing, aging, and sequential injection, can be automatically performed (Figure 9b) [64]. The high pressure/temperature conditions of the microfluidic system can significantly shorten the synthesis time (2 min) of InP QDs and improved the uniformity of the particle size. Yet, in another seminal study, four types of InP/ZnS QDs with different colors (turquoise, yellow, orange, and red) were obtained by optimizing the flow rate and temperature in a microfluidic device [66]. The corresponding quantum yield of turquoise, yellow, orange, and red colored InP QDs was measured and found to be 20%, 42%, 34%, and 37%, respectively. Moreover, different types of QDs, including InP/ZnS, InP/ZnSe, InP/CdS, and InAs/InP were synthesized by employing a microfluidic chip reactor with sub-channels (Figure 9c) [67]. The results showed that the InP/CdS QDs with different shell thickness can be obtained, resulting in the obvious variation of the photoluminescence spectrum (Figure 9d).

The other types of QDs have also been synthesized by microfluidic technology, such as lead sulfide (PbS), lead selenide (PbSe), copper indium sulfide/zinc sulfide (CuInS_2_/ZnS), silver indium zinc sulfide/zinc sulfide (Ag-In-Zn-S/ZnS), and copper indium zinc sulfide/zinc sulfide (Cu-In-Zn-S/ZnS) [131]. Bakr et al. obtained high-quality QDs by separating the nucleation and growth processes in a two-stage microfluidic reactor system [68]. Compared with the single-stage flow reactor and the conventional bulk synthesis, QDs obtained by the two-stage flow reactor showed high fluorescence quantum yield (about 50.6%). Importantly, the microfluidic technology can also be integrated with the on-line fluorescence detection for real-time evaluation of the synthesis process [69]. DeMello and coworkers developed a robust microfluidic platform, which can inhibit the secondary nucleation and lead to the synthesis of copper indium sulfide/zinc sulfide (CuInS_2_/ZnS) QDs. The optical detection system was installed in the microfluidic device, to monitor the reaction parameters before and after the formation of the shells [120].

#### 2.2.4. Microfluidic Synthesis of MOFs

As a kind of porous organic-inorganic hybrid materials and composed of metal clusters or ions, MOFs are widely used in hydrogen storage [132], gas adsorption and separation [133], drug delivery [134], MRI contrast agents [135], biochemical sensors [136], and catalysts [137]. However, the development of MOFs is generally limited by their uncontrollable shape and particle size [22]. Microfluidic technology has been considered as a new avenue to solve the above problems.

**Figure 10:**
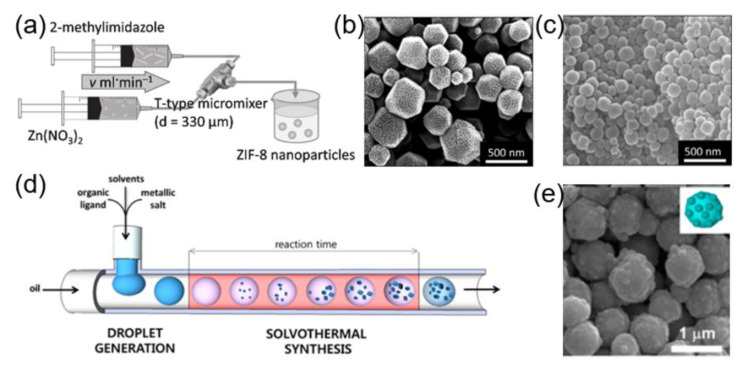
(**a**) Experimental setup for the synthesis of ZIF-8 nanoparticles by using a T-type micromixer. SEM images of ZIF-8 nanoparticles obtained at flow rates of (**b**) 1 mL min^−1^ and (**c**) 30 mL min^−1^ (reproduced from [70], with permission from the Elsevier, 2013). (**d**) Schematic representation of the general micro-chemical process for MOFs. (**e**) Scanning electron microscopy (SEM) image of Fe_3_O_4_@ZIF-8 nanoparticles (reproduced from [138], with permission from the American Chemical Society, 2013).

Mae and coworkers reported the successful fabrication of zeolitic imidazolate framework-8 (ZIF-8) nanocrystals at room temperature, by using T-type microfluidic equipment (Figure 10a) [70]. The results demonstrated that the particle size and morphology of nanocrystals could be controlled by varying the ratio of dimethylimidazole to zinc ions (Figure 10b,c). Besides, different types of MOF-based nanostructures, including Zn_4_O(1,4-benzenedicarboxylate)_3_ (MOF-5), metal–organic framework-3 (IRMOF-3), and UiO-66 (Zr-BDC MOF), have been synthesized in a T-shaped microchip string, by using organic solvents containing ligands and metal precursors as a dispersed phase and silicone oil as a continuous phase (Figure 10d) [138]. Meanwhile, the core-shell structured Fe_3_O_4_@ZIF-8 nanoparticles were obtained by using Fe_3_O_4_ as a precursor (Figure 10e). Since microfluidics can also be used to fabricate nanofibers, Zhao and coworkers performed microfluidic spinning to fabricate core-shell type microfibers, by employing alginate/saline gel as a shell component and copper-vitamin or zinc-vitamin mixture as a core component [139]. The obtained microfibers are long, thin, and flexible, which can be widely used for biomedical applications. To further leverage the potential of microfluidic technology and to preserve the bioactivity of enzymes, ZIF-8 nanoparticles (particle size: ~500 nm) containing enzymes were synthesized by employing a microfluidic gradient mixing approach. It was demonstrated that by continuously varying the concentration of ZIF-8 precursor in the gradient mixing, the surface morphology and microstructure of ZIF-8 could be modulated, which enhanced the activity of the loaded enzymes, as compared to the nonporous ZIF-8 synthesized by conventional bulk method [140]. As a proof-of-principle, glucose oxidase (GOx) loaded ZIF-8 was synthesized by using microfluidic method, which preserved the activity of the enzyme for up to ~98% (the highest record of enzymatic activity for the GOx@MOF composites).

## 3. Progress of Microfluidic Technology for the Surface Modification of Inorganic Nanomaterials

Surface modification has emerged as an effective means to improve the performances of nanoparticles in terms of the dispersibility, surface activity, functionality, and biocompatibility [141,142,143,144,145]. There are numerous methods for the surface modification of nanomaterials, including chemical/electrostatic coating [146], chemical conjugation [147], deposition [148], and microcapsule formation [149]. Unfortunately, traditional surface modification methods often lack accuracy, controllability and repeatability. In contrast, microfluidic technology can be accurately used to realize controllable surface properties of nanomaterials, which is inevitably beneficial for the translation of inorganic nanoparticles to the market. The research on the surface modification of inorganic nanoparticles is mainly divided into two categories: (1) the modification of solid nanoparticles and (2) the modification of porous nanoparticles, which are outlined in the following sections.

### 3.1. Surface Modification of Solid Particles

**Figure 11:**
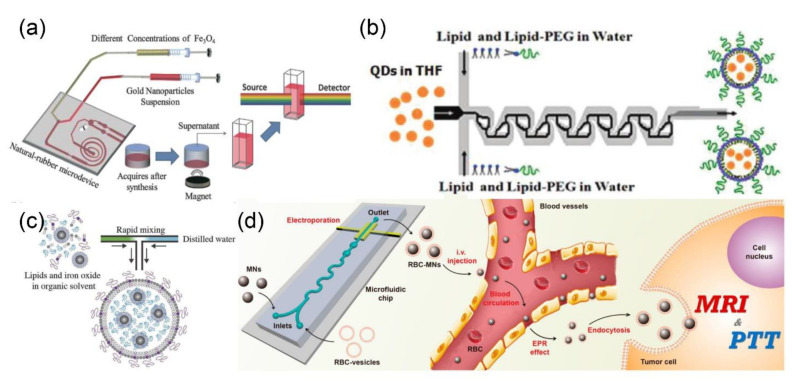
(**a**) Schematic illustration for the synthesis process of Fe_3_O_4_-Au nanoparticles, by using natural-rubber microdevice (reproduced from [95], with permission from the Royal Society of Chemistry, 2015). (**b**) Schematic illustration of the QD-liposome formation in the Tesla mixer (reproduced from [150], with permission from the American Chemical Society, 2010). (**c**) Schematic illustration of the rapid mixing of lipids and IONPs in microfluidic device (reproduced from [151], with permission from the Royal Society of Chemistry, 2017). (**d**) Microfluidic electroporation-facilitated the synthesis of cell membrane coated magnetic nanoparticles for enhanced imaging-guided cancer therapy (reproduced from [153], with permission from the American Chemical Society, 2017).

Different solid nanoparticles including metal, metal oxide and QDs have been modified by using microfluidic approaches for an array of biomedical applications. For example, the surface of Fe_3_O_4_ nanoparticles (size, ~10 nm) was decorated with gold nanoparticles (size, ~4 nm) by using a microfluidic device (Figure 11a) [95]. Neither organic solvents nor surfactants were used in the synthesis, which makes them amenable for clinical applications. Karnik and coworkers realized the self-assembly of monodispersed lipid polymers and QD nanoparticles in a single mixing step, by using microfluidics flow focusing technology (Figure 11b) [150]. The rapid mixing in the microfluidics system led to the formation of QDs@lipid nanoparticles with relatively narrow size distribution. The physical and chemical properties of the prepared composite nanoparticles, such as the particle size (35–180 nm) and the Zeta potential (−10 mV to +20 mV), can be controlled by simply varying the composition and concentration of the precursor solution. Microfluidic technology can also afford functional nanomaterials. In a recent study, hydrophobic iron oxide nanoparticles (IONPs) were encapsulated into liposomes (Figure 11c) [151]. The average number of the encapsulated IONPs was approximately 40 times higher than that of the hybrid particles synthesized by the bulk method. These hybrid nanoparticles could be used as MRI contrast agents for liver imaging. More importantly, other types of inorganic nanoparticles, such as gold and QDs, could also be introduced into liposomes by employing similar microfluidic approach. For instance, both IONPs and paclitaxel were encapsulated into poly(L-lactide-co-glycolide) (PLGA) nanoparticles by using the microfluidic method [152].

Since cell membrane-cloaked nanoparticles possess prolonged circulation in vivo, as well as tumor tissue accumulation potential, microfluidic technology has also been investigated to fabricate these types of nanostructures. In a seminal study, Liu and coworkers employed electric pulses to promote the penetration of magnetic nanoparticles into red blood cell membrane-derived vesicles (RBC vesicles) during the microfluidic synthesis, which has highly shortened the reaction time as compared to the conventional extrusion method (Figure 11d) [153]. The obtained nanoparticles have the potential to be used as MRI contrast agents and beacons for the photothermal therapy, to afford imaging-guided cancer therapy.

### 3.2. Surface Modification of Porous Materials

Porous inorganic nanomaterials have gathered considerable research interest in biomedical sector owning to their rich and adjustable nanoporous structure, high specific surface area, and improved physicochemical properties [154,155,156,157]. The porous microstructure provides an indispensable platform for the loading of antitumor therapeutics or other functional agents for tumor therapy [158,159]. In contrast, currently employed porous drug delivery systems generally face several shortcomings, including premature drug leakage and complicated surface modification chemistries [160,161]. Consequently, it is imperative to develop an efficient surface modification strategy to overcome the aforementioned shortcomings.

**Figure 12:**
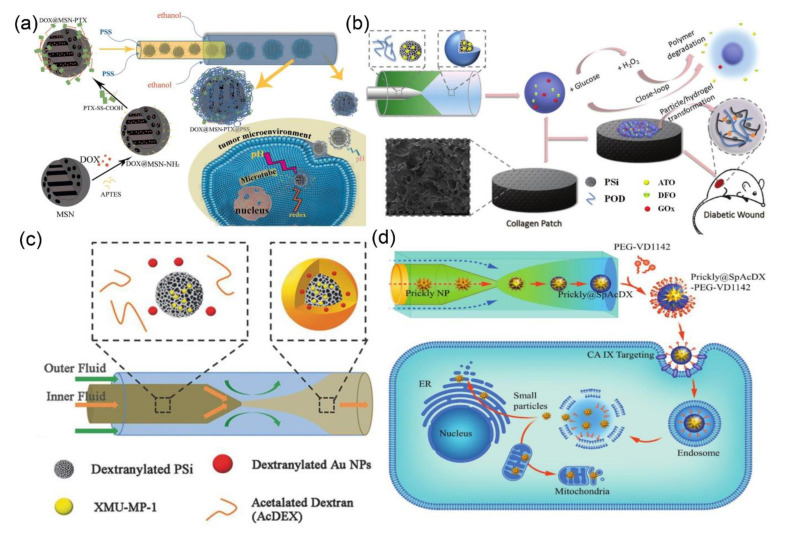
(**a**) Schematic illustration for the synthesis of DOX@MSN-PTX and the microfluidic fabrication of DOX@MSN-PTX@PSS (reproduced from [164], with permission from the American Chemical Society, 2020). (**b**) Schematic illustration for the construction of glucose-responsive delivery system and the orchestrated cascade for diabetic wound care (reproduced from [165], with permission from the Royal Society of Chemistry, 2019). (**c**) Schematic illustration of synthesis procedure of multifunctional nanoparticles by microfluidics (reproduced from [166], with permission from the Wiley-VCH Verlag GmbH & Co, 2018). (**d**) Schematic illustration of the microfluidic synthesis of SpAcDx-coated Zn-CuO nanoparticles, and their specific tumor targeting and anti-proliferative effect (reproduced from [167], with permission from the Wiley-VCH Verlag GmbH & Co, 2017).

Mesoporous silica and porous silicon (PSi) nanoparticles have been hot spots for disease diagnosis and drug delivery for a long time [38,158]. The release kinetics of therapeutic molecules from porous nanoparticles can be controlled by coating stimuli-responsive (e.g., light, heat, temperature, pH, and redox) polymers on the particle surface [162,163]. In these settings, Zhang and coworkers deposited polystyrene sulfonate (PSS) on chemotherapeutic drugs encapsulating MSN by using microfluidic technology (Figure 12a) [164]. The PSS can completely block the open pores of the MSNs and prevent the premature leakage of chemotherapeutic drugs during blood circulation. Moreover, the protonation of PSS occurs in the weakly acidic microenvironment of tumor to accelerate the drug release from MSNs, therefore leading to the tumor-specific drug delivery. Santos’ team has elegantly reported that the surface modification of PSi nanoparticles with dextran-based polymers via the one-step microfluidic self-assembly method, which significantly reduced particle size distribution, improved the surface smoothness, and enhanced the cytocompatibility [38]. More recently, the same group synthesized a reactive oxygen species (ROS)-responsive 4-(hydroxymethyl)-phenylboronic acid pinacol ester/oxidized dextran copolymer (POD) and coated it on the atorvastatin-loaded PSi nanoparticles for diabetic wound healing (Figure 12b) [165]. The degradation of the POD was accelerated by the hydrogen peroxide (H_2_O_2_) to trigger the release of atorvastatin, which was maintained for more than 24 h. Moreover, the multifunctional oxidized dextran nanocarriers consisting of drug-loaded PSi and gold nanoparticles have been synthesized by using a similar approach, which can be used for controlled drug delivery and X-ray computed tomography (CT) imaging for liver failure theranostics (Figure 12c) [166]. Santos et al. have also deposited pH-responsive spermine-modified acetalated dextran (SpAcDx) on the surface of pre-synthesized zinc-doped copper oxide (Zn-CuO) nanoparticles by microfluidic technology for tumor microenvironment-responsive therapy (Figure 12d) [167]. The targeting ligand VD1142 was grafted on a SpAcDx shell to specifically recognize the over-expressed transmembrane protein, carbonic anhydrase IX, in cancer cells. The in vitro results showed that SpAcDx coating shields the Zn-CuO nanoparticles during blood circulation. On the other hand, the SpAcDx coating was collapsed upon entering into the tumor cells, resulting in the exposure of nano-pierces of Zn-CuO nanoparticles that caused severe damage to the endoplasmic reticulum and mitochondria.

## 4. Conclusions and Future Outlook

Microfluidic platforms have shown promising potential to design a myriad of inorganic nanomaterials with an improved trait of physico-chemical properties. Rapid and efficient mixing, as well as simplicity and reversibility, pose microfluidics as an ideal platform for the cost-effective mass production of inorganic nanomaterials with narrow size distribution and high monodispersity than that of the bulk method. Likewise, the application of microfluidic technology for the synthesis of core-shell nanostructures avoids complicated coating and purification steps, in marked contrast to the conventional methods, which encompass separate steps for the nucleation, growth, and modification.

In recent years, although the progress has been made in the synthesis of various types of inorganic nanoparticles (including silica, metal and metal/composite nanoparticles, QDs, MOFs, etc.) and their applications in a wide range of fields; the development of the microfluidic-based synthetic strategy for inorganic nanoparticles is still at its infancy. In order to further promote the rapid development of this technology, several issues need to be carefully addressed, which include, but are not limited to: (1) a comprehensive understanding of the synthesis mechanism of inorganic nanoparticles. At present, the formation processes of inorganic nanomaterials as well as the correlation between particle morphology/structure and reaction conditions in microfluidic scale are not yet fully understood. We look forward to more in-depth investigations of the differences in basic principles between the bulk and microfluidic synthesis. (2) There is few research studies on the effect of liquid evaporation caused by high temperature on the synthesis process of nanomaterials in microfluidic system, which need to be further studied. (3) Increase of the yield of inorganic nanoparticles. One of the advantages of microfluidic technology is that it can accurately process a trace amount of samples, thereby reducing the reagent cost while avoiding the wastage of precious chemicals. However, due to the small channel size and the high hydraulic pressure of the microfluidic device, the current maximum productivity of this technology is only about in grams per hour. In order to meet industrial demands, it is pertinent to develop effective stackable microreactor systems and industrial-scale fluid control devices to achieve a manufacturing efficiency for up to kilograms per hour or even higher. (4) Modulation of the morphology of inorganic nanoparticle. Anisotropic nanoparticles or nanoparticles with different layers have received enormous interest of the scientific community, partly due to their unique morphology and diverse potential applications. However, most of the nanoparticles prepared by using microfluidics are of symmetrical shape. The controllable synthesis of complex shaped nanoparticles based on microfluidic technology has yet to be achieved, and considerable efforts should be made in this field in the near future. (5) Industrial design and development of microfluidic devices. Currently, the manufacturing process of the microfluidic device is very expensive, complicated, and tedious. Companies including Lonza, Corning, and Syrris have conducted groundbreaking research to manufacture robust and flexible microreactors, which provide useful guidance for the industrial development of microfluidics. However, there is still a long way to go to achieve cost-effective yet robust microfluidic devices.

Taken together, microfluidic technology is a promising approach for overcoming the current challenges in the synthesis and modification of inorganic nanomaterials and realizing the well-controlled, cost-effective and reproducible synthesis of inorganic nanoparticles. In the future, with the rapid development of the multi-scale material design, sol-gel chemistry, microfabrication, and microfluidic technology, an array of functional inorganic nanoparticles, with different sizes, shapes, and properties are expected to be realized to meet the growing demand in different fields.

## Figures and Tables

**Table 1:** Inorganic nanomaterials synthesized by microfluidics as well as their structural characteristics, yields, and applications.

Material Type	Microfluidic Systems	Size	Shape	Yield	Applications	References
SiO_2_	single-phase flow	164–321 nm	solid sphere			[23]
SiO_2_	single-phase flow	50–300 nm	solid sphere			[24]
SiO_2_	single-phase flow	53–176 nm	solid sphere	62 ± 6%		[25]
SiO_2_	single-phase flow	200–400 nm	mesoporous (~2 nm) sphere			[27]
SiO_2_	single-phase flow	50–650 nm	hollow; mesoporous sphere			[28]
SiO_2_; SiO_2_-QDs/Fe_3_O_4_	single-phase flow	804 nm	hollow sphere		cell imaging, dye adsorption and drug delivery.	[26]
SiO_2_	single-phase flow	100 nm/ 15 μm	hollow; mesoporous sheet		organics adsorption, protein immobilization, and drug encapsulation	[29]
SiO_2_; SiO_2_-Ag/Fe_3_O_4_	single-phase flow	~130 /1500 nm	mesoporous (~3 nm) fiber	gram-scale	doxorubicin (DOX) loading; 4-nitrophenol reduction	[30]
SiO_2_	single-phase flow	80 × 150 nm	hollow; mesoporous ellipsoid		DOX loading	[31]
Fe_2_O_3_@SiO_2_	single-phase flow	50–350 nm	solid sphere; cube; rod; belt		circulating tumor cell screening	[32]
SiO_2_@TiO_2_	multiphase flow	~250 nm	solid sphere			[33]
SiO_2_@Au	multiphase flow	177–260 nm	solid sphere			[34]
SiO_2_–FITC	multiphase flow	50–350 nm	solid sphere			[35]
SiO_2_–HDDA	multiphase flow	~500 nm	mesoporous patchy			[36]
SiO_2_@Au	multiphase flow	230 nm	solid sphere			[37]
SiO_2_–dextran	multiphase flow	150–400 nm	mesoporous sphere		PTX, SFN, and MTX drug loading	[38]
SiO_2_@Fe_2_O_3_	multiphase flow	~100 nm	solid sphere			[39]
SiO_2_@Au	multiphase flow	~175 nm	solid sphere			[40]
SiO_2_–PEGDA	multiphase flow	100–150 nm (sectional diameter)	macroporous fiber			[41]
SiO_2_–FITC	multiphase flow	10–65 nm	solid sphere			[18]
Au	single-phase flow	15–24 nm	solid sphere		labelling	[42]
Au	single-phase flow	1–2.5 nm	nanoplates		glucose oxidation	[43]
Au	single-phase flow	3–50 nm	nanorods			[44]
Ni	multiphase flow	60–114 nm	solid sphere	11.5 g h^−1^	catalytic hydrogenation of p-nitrophenol to p-aminophenol	[45]
Ni; Ni/SiO_2_	multiphase flow	8.8–15.4 nm	solid sphere	>27 g d^−1^	catalytic hydrodeoxygenation of guaiacol.	[46]
Ag	single-phase flow	~8 nm	solid sphere			[47]
Ag	single-phase flow	edge length: 27–60 nm thickness: 11 nm	solid triangle		LSPR sensing	[48]
Ag	single-phase flow	5–10 nm	solid sphere		4-nitrophenol reduction	[49]
Pd	multiphase flow	2.3 ± 0.3 nm	solid sphere	~10 L d^−1^		[50]
Pd	multiphase flow	~4 nm	nanorods	96.5%	catalytic hydrogenation of styrene	[51]
Rh	multiphase flow	3–8 nm	multipods; cuboctahedra		vapor-phase cyclohexene hydrogenation	[52]
Pt_1_Bi_1_; Pt_1_Bi_2_	single-phase flow	~17 nm; ~33.5 nm	V-shaped nanorods; solid sphere		electrocatalysis	[53]
AuPd	single-phase flow	1–3 nm	nanoclusters		catalytic aerobic oxidation of benzyl alcohol	[54]
AuPd	single-phase flow	1–2 nm	solid sphere		CO oxidation	[55]
Pt–Ni	multiphase flow	6–12 nm	octahedra	20–160 mg h^−1^	oxygen reduction reaction catalysts	[56]
Pd@Pt	multiphase flow	12–20 nm	core-shell; icosahedra		oxygen reduction reaction catalysts	[57]
CdSe	single-phase flow	2–4.5 nm	solid sphere			[58]
CdSe	single-phase flow	3.6–5.4 nm	solid sphere			[59]
CdSe	multiphase flow	narrow size distribution.	solid sphere	40–70%		[60]
CdSe/ZnS	single-phase flow	2.8–4.9 nm	core-shell; solid sphere			[61]
CdSe/CdS/ZnS; CdS/ZnS; CdSeS/ZnS	single-phase flow	~1–5 nm	core-shell; solid sphere			[62]
InP	single-phase flow	~5 nm	solid sphere			[63]
InP	single-phase flow	4 nm	solid sphere			[64]
InP	single-phase flow	2.7 nm	solid sphere	63.1 g d^−1^		[65]
InP/ZnS	single-phase flow	2.8–3.9 nm	core-shell; solid sphere		white-light-emitting diode	[66]
InP/ ZnS; InP/ ZnSe; InP/ CdS; InAs/ InP	single-phase flow	4.1–4.9 nm	core-shell; solid sphere			[67]
PbS	multiphase flow	2–5 nm	solid sphere	2.4–2.5 g h^−1^	photovoltaic device	[68]
PbS; PbSe	multiphase flow	3.8–4.5 nm	solid sphere		fabrication of Schottky solar cells	[69]
ZIF-8	single-phase flow	150–465 nm	larger particles with a polygonal shape; smaller particles with roughly spherical shape			[70]
